# Quillaja saponaria fraction QS-18 as an adjuvant for liposomal seasonal influenza vaccines

**DOI:** 10.1038/s41541-026-01457-1

**Published:** 2026-04-28

**Authors:** Qinzhe Li, Zachary Sia, Yuan Luo, Wei-Chiao Huang, Hilliard L. Kutscher, Haojun Zhu, Joaquin Ortega, Bruce A. Davidson, Jonathan F. Lovell

**Affiliations:** 1 Department of Biomedical Engineering, State University of New York at Buffalo, Buffalo, NY USA; 2 POP Biotechnologies, Buffalo, NY USA; 3 Department of Anatomy and Cell Biology, McGill University, Montreal, QC Canada; 4 Department of Anesthesiology, State University of New York at Buffalo, Buffalo, NY USA; 5 Veterans Administration Western New York Healthcare System, Buffalo, NY USA

**Keywords:** Biotechnology, Drug discovery, Immunology, Microbiology

## Abstract

Saponin fractions from *Quillaja saponaria* (QS) are promising vaccine adjuvants. Among these, QS-21 is a component in several FDA-approved vaccines, but its limited quantity may present a manufacturing bottleneck. While the related saponin, QS-18, is one of the most abundant fractions, its putative toxicity has historically hindered its clinical development. In this study, we formulated QS-18 into a seasonal influenza virus liposomal nanoparticle protein vaccine comprising contemporary, recombinant hemagglutinin and neuraminidase antigens from influenza A (H1N1), influenza A (H3N2), and influenza B (Victoria lineage). This formulation did not exhibit overt QS-18 toxicity in mice or rabbits. In mice and ferrets, QS-18 adjuvanted particles elicited efficacious antibody responses and provided protection against influenza challenges, comparable to analogous QS-21 adjuvanted liposomes. These findings suggest that more study of QS-18 or QS-18-containing QS fractions is warranted for infectious disease vaccine adjuvant formulations.

## Introduction

Vaccine adjuvants are substances added to vaccines to enhance immunogenicity, enable dose-sparing, and promote robust immune responses. Among them, saponin-based adjuvants derived from *Quillaja saponaria* (QS), the Chilean soapbark tree, have shown considerable promise^1^. For instance, the saponin fraction QS-21 is the active component in the clinically successful adjuvant system AS01, used in Shingrix^2^ and Mosquirix^3^ vaccines. In addition, Matrix-M, another Quillaja-derived saponin adjuvant used in the Novavax formulation, also demonstrates clinical efficacy^4^. Mechanistically, saponin-based adjuvants are suggested to enhance the immune response by activating antigen-presenting cells (APCs), leading to downstream T cell activation and the induction of Th1 immune responses^5^, and activating the NLRP3 inflammasome, leading to caspase-1-dependent IL-1β and IL-18 release in antigen-presenting cells^6^.

Several purified saponin fractions are available from QS extract, including, for example, QS-7, QS-17, QS-18, and QS-21^7^. Among these, QS-21 has been by far the most widely used due to its favorable balance of relatively low toxicity and strong in vivo immunogenicity^7^, and it is a well-characterized isomeric mixture of triterpene glycosides for modern vaccine development^5,8,9^. However, QS-21 constitutes only approximately 3.7% of crude QS extract^10^, limiting supply and complicating large-scale manufacturing. In contrast, QS-18 constitutes approximately 35.1% of the QS extract^10^, but might show greater in vivo toxicity compared to QS-21. Due to earlier research suggesting unformulated QS-18 had higher toxicity in mice^7^, the use of QS-18 as an adjuvant appears to have been largely avoided. Liposomes have been used to formulate saponins and can mitigate their toxicity^11,12^.

Influenza virus remains one of the most significant respiratory pathogens in the United States, with an estimated 40 million illnesses, over 470,000 hospitalizations, and more than 28,000 deaths during the 2023-2024 season, according to CDC data^13^. Seasonal influenza vaccinations continue to be an effective method for limiting infection, hospitalizations, and mortality^14^. Consequently, there is a continuous need for more effective seasonal influenza vaccines. Furthermore, because coordinating the influenza vaccine supply chain to meet uncertain seasonal demand remains a significant challenge^15^, the inclusion of effective adjuvants offers a critical advantage by enabling dose-sparing and expanding the available vaccine supply.

Liposomes have been used for many purposes, including vaccine adjuvant delivery, of which potential advantages include solubilization of adjuvants that are hydrophobic or lipid-like, improving delivery to antigen-presenting cells, and enhanced immune responses through improved antigen presentation^16–18^. Previous studies have shown that adjuvanted influenza vaccines are a promising solution for enhancing immune responses^19–22^. We have also validated the use of the QS-21 saponin fraction in cobalt-porphyrin phospholipid (CoPoP) liposomal vaccines^23^. CoPoP itself is a lipid that serves to couple recombinant proteins and peptides to the liposome carrier that has advanced through successful Phase 3 clinical trials^24,25^. We therefore hypothesized that, using the liposomal formulation for the inclusion of QS-18 and QS-21 without chemical modification, thereby avoiding potential alteration in adjuvant activity and regulatory complexity. Further, incorporation of QS-18 with PHAD into a CoPoP nanoparticle vaccine (*C: CoPoP; P: PHAD; Q: QS adjuvant*; CPQ^18^) mitigates intrinsic hemolytic activity while maintaining an adjuvant efficacy comparable to QS-21 adjuvanted nanoparticle vaccine (CPQ^21^). Recombinant His-tagged seasonal influenza HA and NA antigens were then displayed on the liposome surface, enhancing antigen presentation and immunogenicity compared to traditional adjuvants. Subsequent hexaplex CPQ^18^ and CPQ^21^ immunization conferred protection against seasonal influenza challenge in both mouse and ferret models. Our study demonstrates how an abundant saponin, QS-18, with putative safety concerns, can be transformed into a tolerable and effective vaccine adjuvant, and highlights the potential of QS-18 to ameliorate the supply constraints of QS-21 in future vaccine formulations.

## Results

### Hexaplex liposomal vaccine design and biophysical characterization

**Fig. 1:**
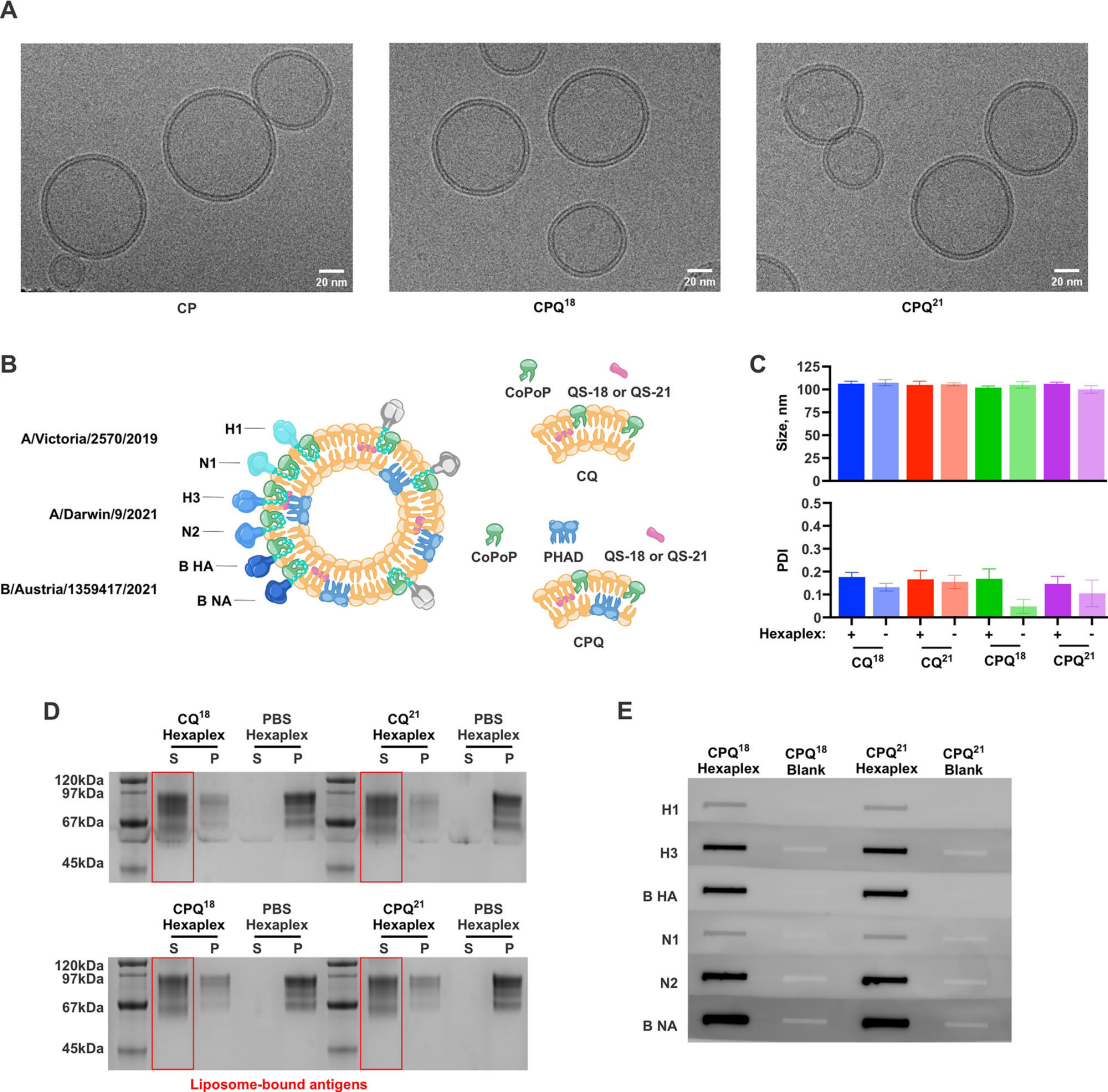
Design and biophysical characterization of CoPoP liposomes containing QS-18 (CPQ^18^) or QS-21 (CPQ^21^). **A** Cryo-electron microscopy (Cryo-EM) image of CoPoP/PHAD liposome (CP), CoPoP/PHAD liposome with QS-18 (CPQ^18^), and CoPoP/PHAD liposome with QS-21 (CPQ^21^). **B** Design of CoPoP liposome displaying hemagglutinin and neuraminidase of the three seasonal influenza strains. (**C**) Size and PDI measured by dynamic light scattering. **D** Ni-NTA binding assay demonstrating binding of six antigens to CoPoP liposomes (hexaplex); the red box indicates the liposome-bound antigens remained in the supernatant. **E** Slot blot assay detecting surface-displayed antigens on CoPoP liposomes using antigen-specific monoclonal antibodies. In (**C**), data are presented as mean ± standard deviation.

The cryo-electron microscopy (Cryo-EM) imaging of the CoPoP/PHAD liposomes following incorporation of QS-18 or QS-21 was first obtained (Fig. 1A). Intact spherical vesicles with clearly defined lipid bilayers were observed and showed no detectable disruption of liposome bilayer or vesicle morphology. To anchor antigens to the liposome surface via cobalt chelation, a C-terminal His-tag was introduced to all recombinant antigens. We then coupled a hexaplex mixture of seasonal recombinant hemagglutinin (HA) and recombinant neuraminidase (NA) antigens derived from A/Victoria/2570/2019 (H1N1)pdm09, A/Darwin/9/2021 (H3N2), and B/Austria/1359417/2021 (B/Victoria lineage; B-Vic), per WHO strain recommendation guidelines, as previously described^23^, to CPQ^18^ or CPQ^21^ at a 3:1 CoPoP to antigen mass ratio (Fig. 1B). Dynamic light scattering (DLS) analysis showed that both the hexaplex CPQ^18^ and CPQ^21^ nanoparticles were approximately 100 nm in size with a PDI below 0.3, indicating a uniform particle distribution (Fig. 1C). To confirm high binding efficiency, we used Ni-NTA beads to competitively bind and separate antigens from the liposome (Fig. 1D). The results showed that the majority of the antigen remained in the supernatant (“S” lane) with the liposomes, with minimal amounts detected in the bead pellet (“P” lane), confirming efficient antigen capture. Finally, to ensure correct antigen presentation on the liposome surface, we performed a slot blot using monoclonal antibodies specific to each of the six antigens. The chemiluminescence signal for all antigens indicated that they were correctly displayed and accessible for antibody binding (Fig. 1E). Once bound to the liposomes, the His-tag of the proteins became inaccessible to an anti-His tag antibody, likely due to the His tag anchoring in the membrane (Supplementary Fig. S1). In Phase 2 testing, a His-tagged RBD antigen did not elicit significant anti-His tag responses in humans^26^. Together, these data show that liposomal surface presentation of His-tagged antigens is possible on the CoPoP/PHAD liposomes with the incorporation of either QS-18 or QS-21.

### Evaluation of QS-18 and QS-21-containing hexaplex liposomal vaccine tolerability in mice

**Fig. 2:**
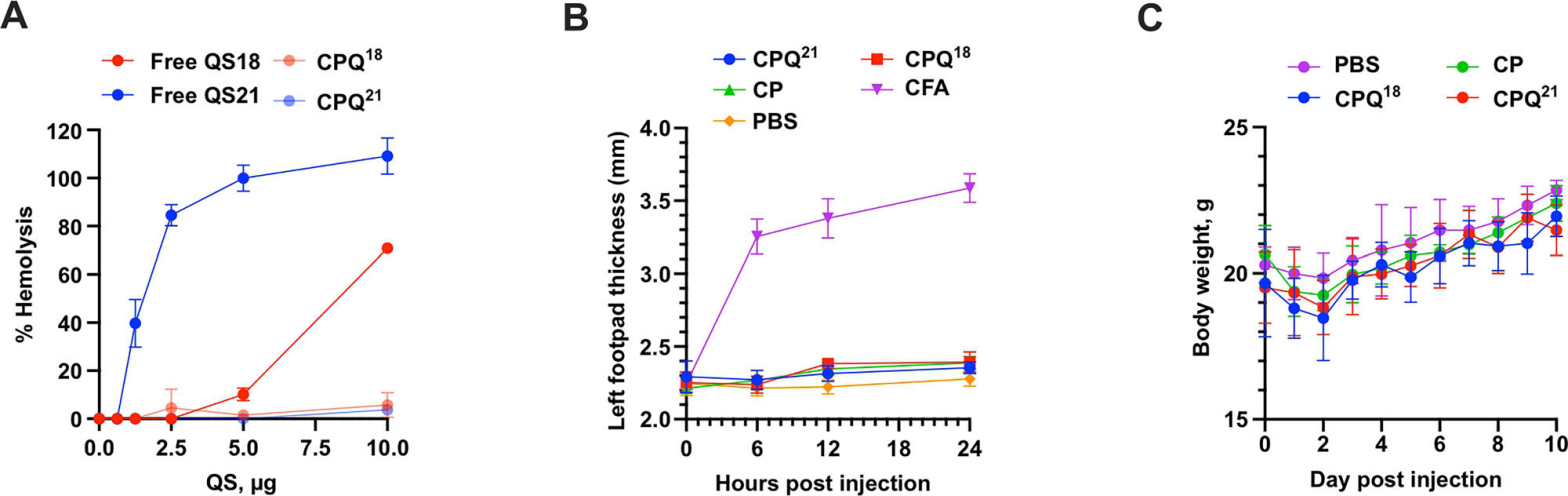
CoPoP/PHAD liposomes formulated with QS-21 or QS-18 are well tolerated in mice. **A** In vitro hemolysis induced by free (unformulated) QS-21 or QS-18, or when formulated with CoPoP liposomes. **B** BALB/c mice left footpad thickness change after receiving CoPoP liposome formulated with 2.16 µg of QS-21 or QS-18, compared to CoPoP/PHAD liposome only and CFA; *n* = *4*. **C** Daily body weight of mice was monitored following intramuscular administration of the same formulation; *n* = *5*. Data are presented as mean ± standard deviation.

Given the saponin adjuvants’ property that potentially interacts with cholesterol and disrupts membranes^12,27,28^, turkey red blood cell (RBC) hemolysis was performed as an in vitro assay to evaluate hemolytic activity (Fig. 2A). As expected, both QS-18 and QS-21 in their free form exhibited significant hemolytic activity. In contrast, when incorporated into CP liposomes, which contain cholesterol, both saponins induced significantly less hemolysis. This is likely because the saponins are incorporated within the liposomal bilayer, where their interaction with cholesterol mitigates their membrane-disrupting effects before contacting the cellular membrane. Next, since AS01 adjuvant, which contains QS-21, is thought to be relatively reactogenic in some cases^29,30^, we assessed footpad swelling to determine how QS-18 would compare to QS-21 embedded in CP liposomes. Compared to hexaplex antigens formulated with Complete Freund’s Adjuvant (CFA), hexaplex CPQ^18^ induced only transient mild swelling at 12 h post-injection (*p* = 0.0180 vs the PBS group), while hexaplex CPQ^21^ did not cause detectable inflammation at the injection site. In contrast, CFA-formulated vaccines caused pronounced inflammation and swelling (Fig. 2B). 4-6 weeks old BALB/c mice were immunized with a single dose of hexaplex vaccine, and body weights were monitored for 10 days post-injection (Fig. 2C). Blood was collected on day 10 for a complete blood count (CBC) and serum chemistry analysis (Supplementary Figs. S2 and S3).

### Evaluation of QS-18 and QS-21-containing liposomal vaccine tolerability in rabbits

**Fig. 3:**
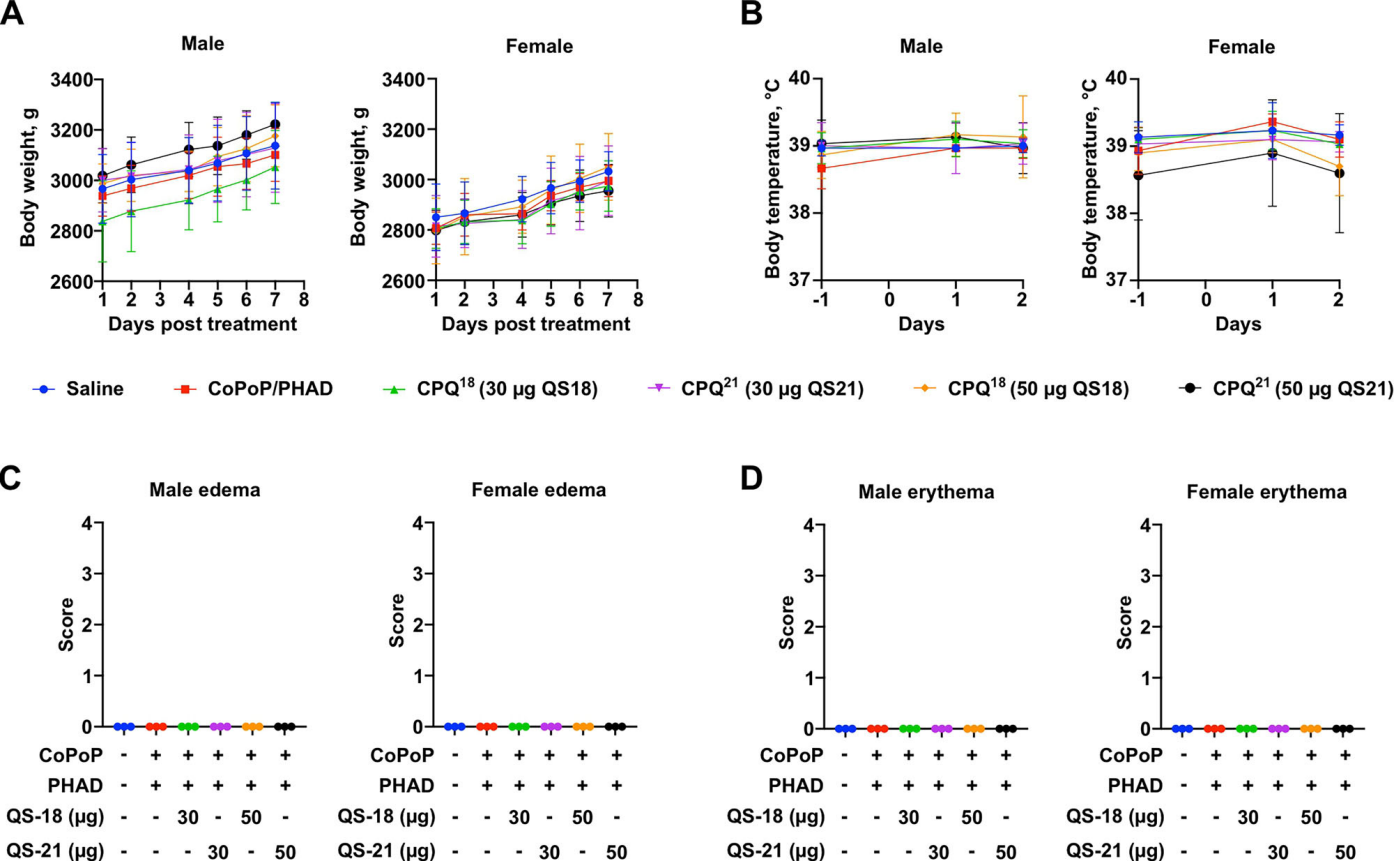
Tolerability of a single intramuscular dose of QS-18 and QS-21 in New Zealand white rabbits. CPQ^18^ or CPQ^21^ formulations containing different doses of QS-18 or QS-21 were administered via intramuscular injection; *n* = *6*, 3 males and 3 females. **A** Body weights and **B** body temperatures were monitored post-treatment. **C** Local edema and **D** local erythema scores following treatment. Data are presented as mean ± standard deviation.

We next conducted a formal tolerability study in male and female New Zealand white rabbits via intramuscular injection. The high-dose group received 50 µg of QS-18 or QS-21 with 125 µg CoPoP and 50 µg PHAD. The low-dose group received 30 µg of QS-18 or QS-21 with 75 µg CoPoP and 30 µg PHAD. All treatment groups were compared to the control group that received sterile saline and the vehicle group that received 125 µg CoPoP and 50 µg PHAD. Immunization with CoPoP formulations containing either QS-18 or QS-21 at doses up to 50 µg was well-tolerated, with no effect on mortality, body weight (Fig. 3A), or body temperature (Fig. 3B). Furthermore, dermal Draize scoring assessments showed no injection site abnormalities (Figs. 3C, D). We did observe a mild, transient increase in fibrinogen in the high-dose groups, which resolved by day 8 (Supplementary Fig. S4). Other non-adverse changes in clinical chemistry and coagulation were noted on day 3 but also resolved on day 8, consistent with the expected acute-phase protein response following administration of an adjuvanted formulation (Supplementary Figs. S5–S8). Collectively, these findings indicate that a single intramuscular injection of CP liposomes containing QS-18 or QS-21 was well tolerated in male and female New Zealand white rabbits at doses up to 50 µg.

### Immunogenicity and functional antibody responses elicited by the hexaplex liposomal vaccine

**Fig. 4:**
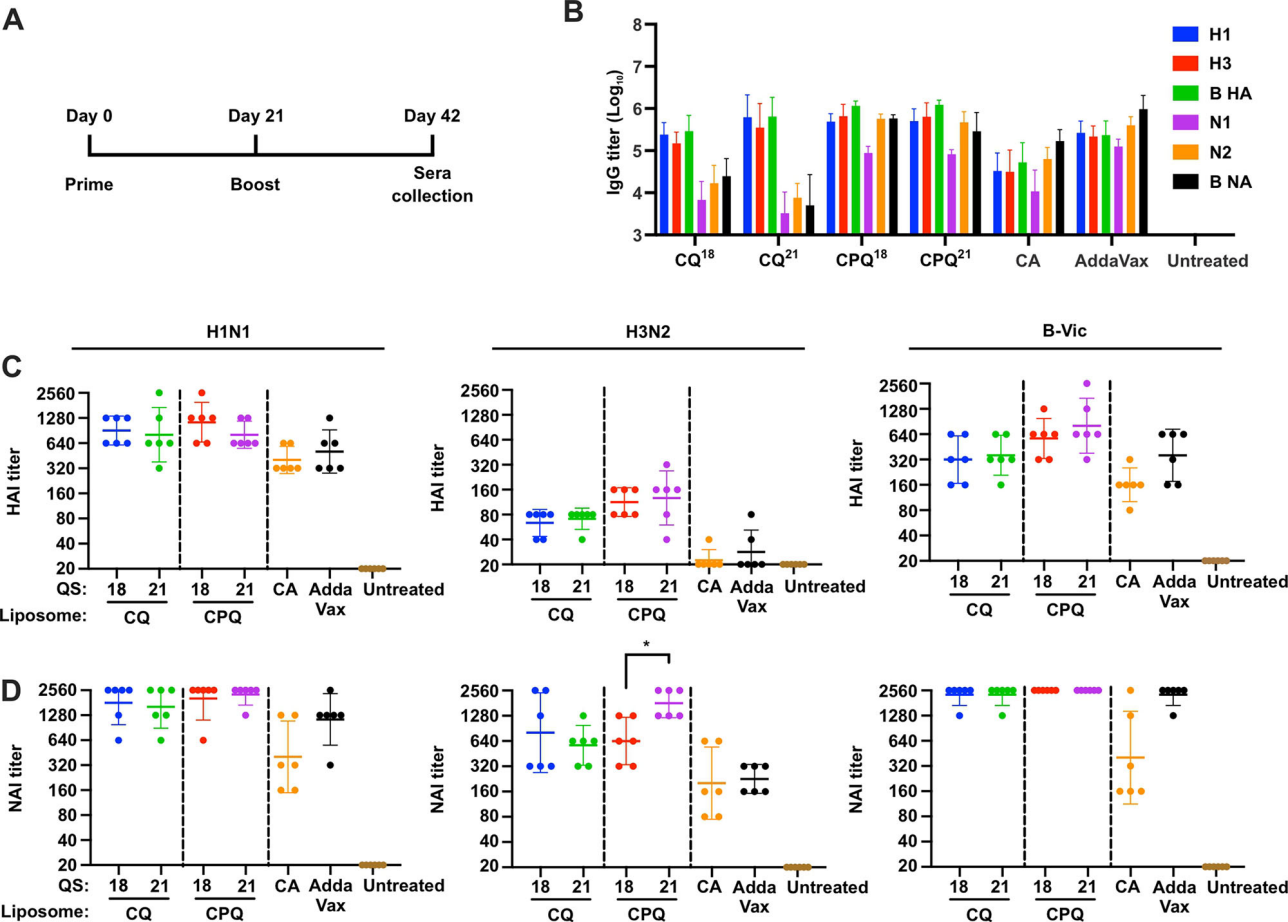
Functional antibody responses induced by hexaplex CPQ^18^ are comparable to those induced by hexaplex CPQ^21^. **A** Mice received prime and booster 3 weeks apart, and sera were collected on day 42; *n* = *6*. **B** Binding antibody response to the HA or NA ectodomain. **C** Functional antibody level assessed by hemagglutination inhibition assay in response to A/Hawaii/66/2019 (H1N1), A/Perth/16/2009 (H3N2), and B/Washington/02/2019 (B-Vic). **D** functional antibody level for neuraminidase inhibition assessed by colorimetric enzyme-linked lectin assay, in response to A/Hawaii/66/2019 (H1N1), A/Perth/16/2009 (H3N2), and B/Washington/02/2019 (B-Vic). Data are presented with geometric mean with 95% CI. Significances in (**B**) were analyzed by a two-way ANOVA followed by Tukey’s multiple comparisons test. Significances in (**C**) and (**D**) were analyzed by a one-way ANOVA followed by Tukey’s multiple comparisons test. *P < 0.05; ns, not significant.

4–6-week-old ICR mice were immunized with two doses of the hexaplex vaccine, with immunization on day 0 and day 21, and serum collection on day 42 (Fig. 4A). This dosing regimen aligns with our prior work using CPQ^21^ liposomes^23,31^. To investigate the contribution of PHAD to the immune response, we also included a control group immunized with a liposome lacking both PHAD and QS-adjuvants. ELISA analysis indicated no difference in IgG titers against all antigens between the CPQ^18^ and CPQ^21^ immunized groups on day 42 (Fig. 4B). Functional assays, including hemagglutination inhibition (HAI) assays and neuraminidase inhibition (NAI) assays, were performed next to confirm the potent and comparable immune response. Both QS-18 and QS-21 adjuvanted hexaplex CP were able to induce significant neutralizing antibodies against influenza H1N1, influenza H3N2, and influenza B-Victoria strains. No significant difference between the HAI titer of QS-18 and QS-21 adjuvanted hexaplex CP immunized groups for all three strains, indicating that the adjuvant potency of QS-18 and QS-21 is comparable in inducing HA-inhibiting antibodies (Fig. 4C). Similarly, NAI titers were also comparable for the H1N1 and B-Victoria strains’ NA-inhibiting antibodies, though the CPQ^21^ group showed a higher NAI titer for H3N2 strain NA-neutralizing antibodies compared to the CPQ^18^ group (Fig. 4D). Overall, these data demonstrate that QS-18 immunogenicity was comparable to QS-21, with both formulations generating high titers of functional and neutralizing antibodies against seasonal influenza viruses in mice.

### Protection against lethal influenza challenge following hexaplex liposomal vaccination in mice

**Fig. 5:**
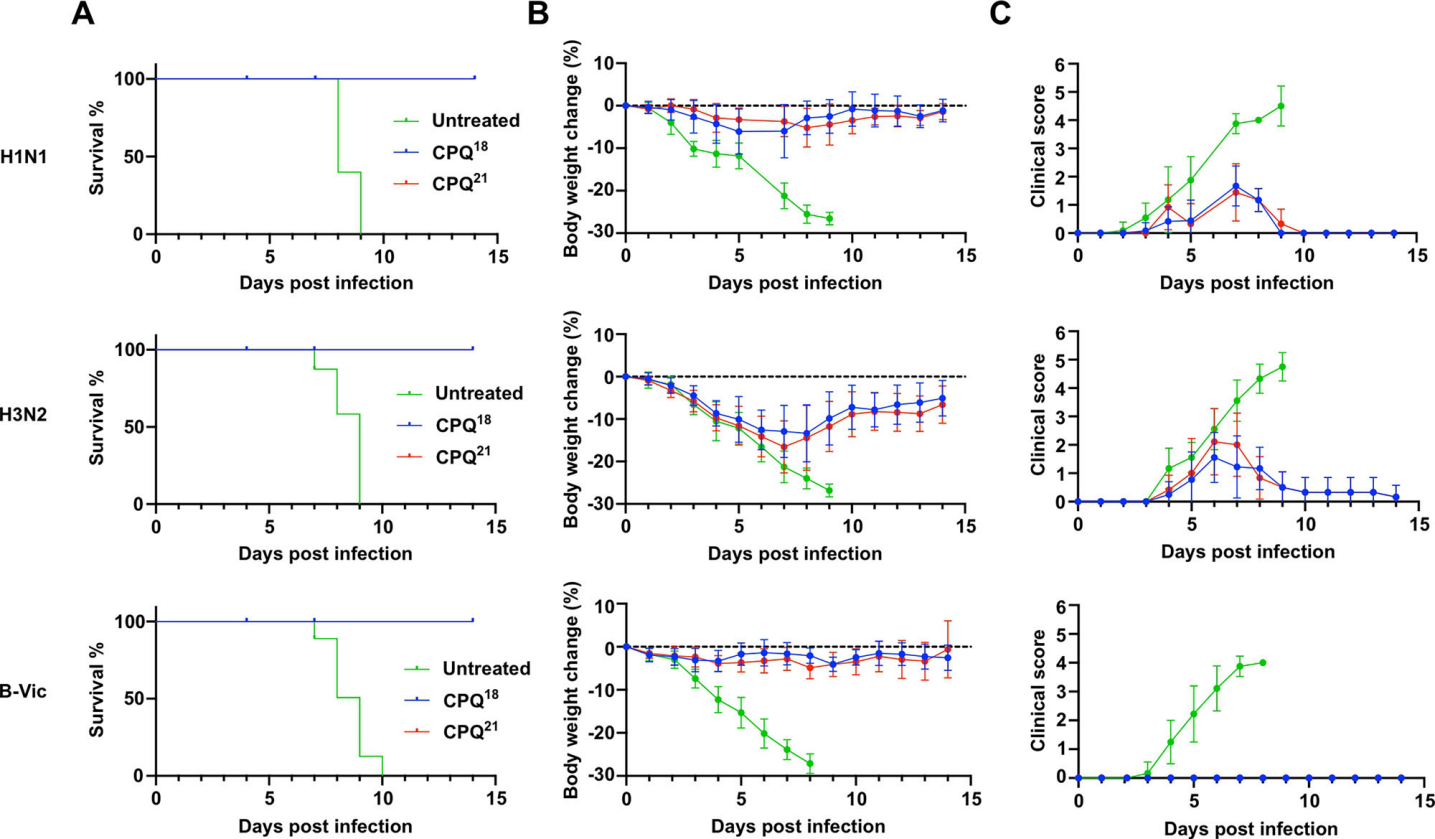
Hexaplex CPQ^18^ and CPQ^21^ conferred protection against lethal influenza challenge *in vivo.* 4–6 weeks old BALB/c mice received two doses of hexaplex CPQ^18^ or CPQ^21^ three weeks apart, followed by intranasal inoculation of mouse-adapted A/California/04/2009 (H1N1), mouse-adapted A/Hong Kong/1/1968 (H3N2), or mouse-adapted B/Malaysia/2506/2004 (B-Vic) on day 42. **A** Survival outcome. **B** Post-challenge body weight change. **C** Post-challenge clinical score. In (**B**) and (**C**), data are presented as mean ± standard deviation.

Next, BALB/c mice were immunized with hexaplex CPQ^18^ or CPQ^21^ and challenged with mouse-adapted influenza in vivo. A 14-day survival outcome of mouse-adapted H1N1, H3N2, and B-Vic influenza virus challenge demonstrated complete protection from hexaplex CPQ^18^ or CPQ^21^ immunization (Fig. 5A). The significant body weight loss was observed for all mice during the H3N2 challenge from 0 to 7 days post infection (dpi), and recovered after 7 dpi for immunized mice, while unvaccinated control mice weight loss reached a critical point (Fig. 5B). This is likely due to the relatively low sequence identity between the immunized H3N2 HA/NA and challenge H3N2 HA/NA, which are both ~84% match. In contrast, both immunizing HA and NA of H1N1 and B-Vic shared ~94% sequence identity with the challenge strain. Consequently, the immunization tends to generate more strain-matched functional antibody responses, which protect mice from lethal challenge in vivo. Lastly, we monitored and recorded the clinical score based on the behavior of mice after the challenge. Mice showed significantly lower morbidity signs after being immunized with hexaplex CPQ^18^ or CPQ^21^ compared to the unvaccinated group (Fig. 5C).

### Reduction of viral burden following hexaplex liposomal vaccination in mice

**Fig. 6:**
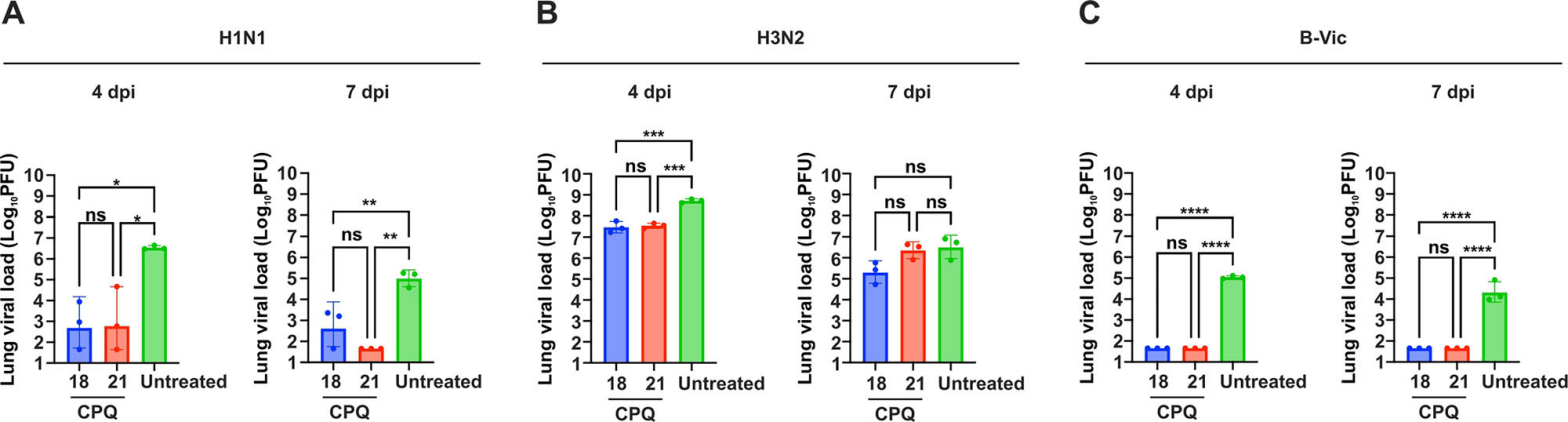
Hexaplex CPQ^18^ and CPQ^21^ reduced pulmonary viral burden following lethal influenza challenge. Lung viral load of mice challenged by (**A**) mouse-adapted A/California/04/2009 (H1N1); (**B**) mouse-adapted A/Hong Kong/1/1968 (H3N2); and (**C**) mouse-adapted B/Malaysia/2506/2004 (B-Vic) at 4 and 7 dpi; *n* = *3*. Data are presented as the geometric mean ± geometric standard deviation. Significances were analyzed by a one-way ANOVA followed by Tukey’s multiple comparisons test. *P < 0.05,**P < 0.01,***P < 0.001,****P < 0.0001; ns, not significant.

To further evaluate viral replication following the challenge, lung viral loads were measured at 4 and 7 dpi, following the same immunization schedule mentioned in Fig. 4A. Compared to the unvaccinated group, both hexaplex CPQ^18^ and CPQ^21^ were able to significantly reduce the lung viral load at 4 and 7 dpi (Figs. 6A, C). However, despite both hexaplex CPQ^18^ and CPQ^21^ being able to reduce lung viral load at 4 dpi, there were still substantial amounts of flu load detected, which likely contributed to the body weight loss during mouse-adapted A/Hong Kong/1/1968 (H3N2) in Fig. 5B (Fig. 6B). Overall, these data confirm that both hexaplex CPQ^18^ and CPQ^21^ provided comparable protection against lethal influenza challenge by enabling effective control of viral replication in the lungs.

### Protection against A/Wisconsin/67/2022 (H1N1) challenge following hexaplex liposomal vaccination in ferrets

**Fig. 7:**
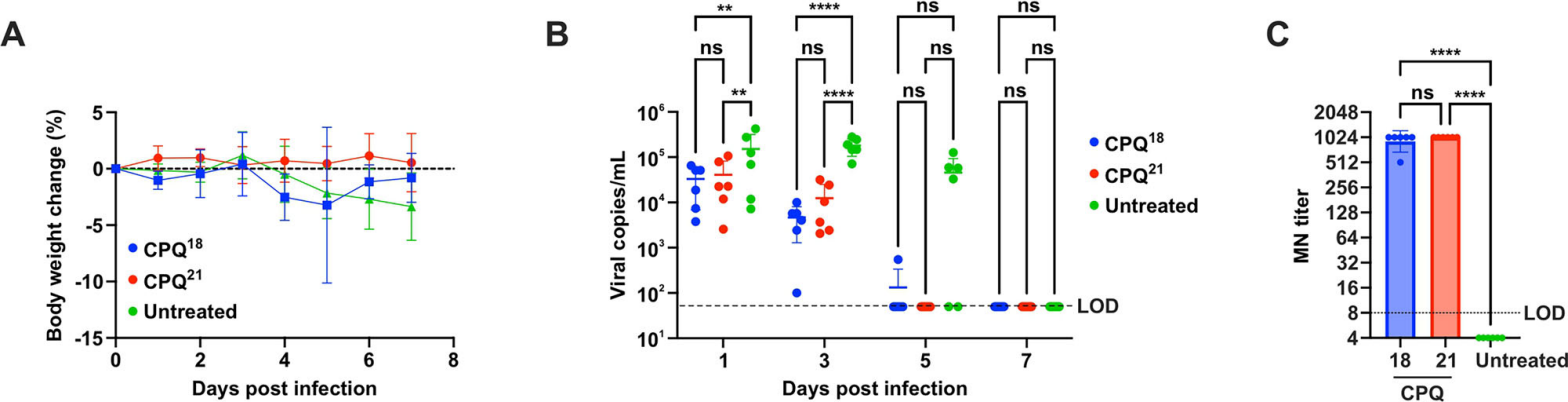
Hexaplex CPQ^18^ and CPQ^21^ were immunogenic and protected ferrets against A/Wisconsin/67/2022 (H1N1) challenge. Ferrets received two doses of hexaplex CPQ^18^ or CPQ^21^, three weeks apart, then challenged by A/Wisconsin/67/2022 (H1N1) on day 42. **A** Body weight changes post-viral infection. **B** Viral load was detected in the nasal wash post-infection. **C** microneutralization (MN) titer against A/Wisconsin/67/2022 (H1N1) detected on day 39 post-immunization, before the viral infection. Data in (**A**) and (**B**) are presented as the mean ± standard deviation. Data in (**C**) are presented as the geometric mean with 95% CI. Significances were analyzed by a one-way ANOVA followed by Tukey’s multiple comparisons test. **P < 0.01; ****P < 0.0001; ns, not significant.

Finally, we evaluated the efficacy of hexaplex CPQ^18^ or CPQ^21^ in protecting against A/Wisconsin/67/2022 (H1N1) infection in a ferret model, the gold standard for human influenza vaccine development. Following the challenge, no animals lost significant body weight (Fig. 7A). To assess the impact on viral replication, qPCR was used to detect viral load in the nasal wash at 1, 3, 5, and 7 dpi. Notably, at 1 and 3 dpi, hexaplex CPQ^18^ and CPQ^21^ significantly reduced viral copies compared to the control group, while no viral copies difference was observed between the hexaplex CPQ^18^ and CPQ^21^ groups (Fig. 7B). In addition, pre-challenge serum collected from both hexaplex CPQ^18^ or CPQ^21^ immunized groups on day 39 before the viral challenge showed high microneutralization (MN) titers against A/Wisconsin/67/2022 (H1N1), likely contributed to the reduced viral burden in the infection (Fig. 7C). Together, these data suggest that both hexaplex CPQ^18^ and CPQ^21^ are comparably effective, generating a potent neutralizing antibody response that provides early protection against H1N1 infection in the ferret model.

## Discussion

While QS-21 has been extensively evaluated in human vaccines, including as a component of the AS01 liposomal adjuvant^2,3^ and as a component of the purified, mixed QS fraction-based formulations such as Matrix-M^4^, QS-18, as one of the most abundant Quillaja fractions, has been neglected because of its putative toxicity compared to QS-21. However, the precise mechanism of QS-18 and QS-21 toxicity is still not fully clear. Previous studies have suggested that the toxicity of QS-based adjuvants was likely related to the interaction between cholesterol, a major component of animal cell membranes that maintains the membrane fluidity and structural integrity^32^. Therefore, a goal of this study was to assess a formulation that mitigates the intrinsic toxicity of QS-18 while preserving its adjuvant effects, aiming for a safety and efficacy profile comparable to a QS-21 adjuvanted vaccine.

QS-18 is an amphipathic triterpenoid glycoside composed of a hydrophobic sapogenin core linked to hydrophilic oligosaccharide chains, which allows partitioning at lipid-water interfaces. Our prior data demonstrated that both QS-18 and QS-21 spontaneously associated with cholesterol-containing liposomes, although the incorporation percentages depend on the cholesterol:saponin ratio^33^. This behavior is consistent with prior reports describing cholesterol-dependent interactions of Quillaja saponins^12,27,28^, and such incorporation attenuates membrane-disruptive and hemolytic activity while preserving adjuvant function without requiring additional chemical modification of the saponin. QS-21 shares similar amphipathic and cholesterol-binding properties and is therefore likely to incorporate into the lipid bilayer through a similar mechanism, although structural differences may influence the intrinsic cytolytic potency. Consequently, both saponins exhibited reduced hemolytic activity and no severe local reactogenicity (Fig. 2), supporting improved tolerability of the CoPoP liposomal formulation, while the detailed molecular basis of bilayer incorporation remains for further investigation.

To further evaluate the potential safety concerns, we performed toxicity studies of both formulations in the mouse and rabbit models, which demonstrated the tolerability of QS-18 and QS-21 as CoPoP liposome adjuvant. We evaluated the local reactogenicity and inflammatory response of hexaplex CPQ^18^ formulations through footpad injection with BALB/c mice, with mild swelling at 12 h post-injection. Mice that received hexaplex CPQ^18^ formulations through intramuscular injection showed no significant body weight loss after 10 days compared to the PBS group. To further assess safety, the tolerability of QS-18 was evaluated in a rabbit model, a standard species for vaccine toxicology studies. Rabbits received QS-18 or QS-21 at doses up to 50 µg with CoPoP and PHAD liposome showed no effect on mortality, dermal Draize observation, body weight changes, body temperatures, or clinical pathology and hematology. However, elevated levels of C-reactive protein (CRP) were detected in males receiving CP containing 50 µg of QS-18 and in both sexes receiving 50 µg of CP containing QS-21, relative to CP vehicle controls (Supplementary Fig. S5 and S6), consistent with an acute-phase response following adjuvant administration. Collectively, these data suggested our formulation successfully mitigates the toxicity of QS-18, but further mechanistic studies and dose adjustment will be required to optimize the balance of safety and efficacy for future clinical development.

Notably, we observed comparable efficacy of hexaplex CPQ^18^ to CPQ^21^. The synergistic effect of HA and NA was also observed in the CPQ^18^ formulation. Flublok, an FDA-approved recombinant influenza vaccine, contains only recombinant HA as the antigen^34^, as HA-specific antibodies can neutralize the virus by blocking its entry into host cells^35^. In contrast, NA-specific antibodies typically do not prevent viral entry but inhibit viral egress, thereby limiting disease severity^36^. In our previous research, mice immunized with HA only received substantial protection, while immunization with both HA and NA provided complete protection against lethal H1N1 viral challenge, likely due to the synergistic effect of HA- and NA-specific antibodies to prevent both viral entry and spread. Ferrets are a well-established model for preclinical influenza vaccine studies due to their similarities to humans in respiratory tract physiology and immune response. In this model, the hexaplex CPQ^18^ was able to induce comparable neutralizing antibodies against the A/Wisconsin/67/2022 (H1N1) to those induced by CPQ^21^. This response corresponded with the reduction of the viral burden during the early viral infection period.

There are several limitations of this study. In the H3N2 mouse challenge model, although hexaplex CPQ^18^ and CPQ^21^ immunization showed complete protection (Fig. 5), residual viral burdens were detected in the lung at 4 dpi (Fig. 6), likely due to the antigenic difference between the A/Darwin/9/2021 (H3N2) antigen and the historical mouse-adapted A/Hong Kong/1/1968 (H3N2). Another limitation is that only a limited safety assessment was performed. While significantly reduced hemolytic activity was observed following liposomal incorporation, more toxicity evaluations, including comparative analysis of membrane interactions and downstream inflammatory responses, are necessary to better define the safety profile of QS-18 and QS-21. The data presented here are insufficient to rule out that QS-18 would not have greater reactogenicity or toxicity issues in humans, so more robust testing is required.

In summary, our study demonstrated that the QS-18-adjuvanted CP platform is well tolerated in both mice and rabbits, confers complete protection against lethal influenza challenge in mice, and prevents early viral infection in ferrets, highlighting the potential of QS-18 as an effective adjuvant for highly-demanded seasonal influenza vaccine development when formulated with the CP platform. While the clinical success of the QS-21 adjuvant is well-established, its scarcity presents a major challenge for vaccine production. Our work demonstrated the comparable immunogenicity of QS-18 when it is formulated with the CP liposome platform, in which the interchangeability not only offers flexibility in vaccine design but also promises to alleviate the significant supply chain pressure on QS-21.

## Methods

### Material

The following vaccination antigens were obtained from Nexelis (Laval, Quebec, Canada) as previous described^23^: A/Victoria/2570/2019 (H1N1) HA (Lot# NL2203M); A/Victoria/2570/2019 (H1N1) NA (Lot# NL2203F); A/Darwin/9/2021 (H3N2) HA (Lot# NL2203L); A/Darwin/9/2021 (H3N2) NA (Lot# NL2204D); B/Austria/1359417/2021 (B-Vic) HA (Lot# NL2204G); B/Austria/1359417/2021 (B-Vic) NA (Lot# NL2204C). Additionally, recombinant H1 and N1 of A/Wisconsin/67/2022 (H1N1), recombinant H3 and N2 of A/Darwin/6/2021 (H3N2), and recombinant B-HA and B-NA of B/Austria/1359417/2021 were obtained from Kinnakeet Biotech (Brandermill, Virginia, United States).

### Liposome preparation

DOPC, cholesterol (CHOL), CoPoP, and 3D6A-PHAD (PHAD) were combined at a mass ratio of 20:5:1:0.4. Liposomes were generated by ethanol injection followed by nitrogen-driven extrusion in phosphate buffered saline (PBS) at 55 °C. The suspension was sequentially extruded through 200 nm, 100 nm, and 80 nm polycarbonate membranes (10 passes each) and then dialyzed against PBS three times over 24 h. QS-18 or QS-21 (1 mg mL⁻¹ stock) was subsequently incorporated into dialyzed CP liposomes to achieve a final ratio of DOPC:CHOL:CoPoP:PHAD:QS-18 or QS-21 = 20:5:1:0.4:0.4. The concentration of CPQ^18^ and CPQ^21^ liposomes was standardized to 320 µg mL⁻¹ based on CoPoP content. Control CP liposomes were prepared identically but without QS-18 or QS-21. CoPoP liposomes without PHAD (CA) were prepared in the same manner. All liposome formulations were stored at 4 °C until use.

### Cryo-electron microscopy

Before applying the samples, C-flat 2/2-2Cu-T grids were washed with chloroform for 2 h and treated with a negative glow discharge in air using a PELCO easiGlow system (Ted Pella, Inc.) at 10 mA for 15 s before freezing. Then, 3.5 μL of CP, CPQ^18^, or CPQ^21^ samples was applied to the grid and let sit for 60 s before it was blotted once and plunged into liquid ethane using a Vitrobot Mark IV (ThermoFisher Scientific). The Vitrobot was set to 4 °C, 100% relative humidity, blot force +1, blot time 2-3 s, and drain time 0.5 s.

Data were acquired using SerialEM software on the Titan Krios electron microscope at FEMR-McGill, operated at 300 kV. The sample was kept at −190 °C during imaging. Images were collected with a Gatan K3 direct electron detector equipped with a Bioquantum imaging filter at defocuses between −1.5 to −2.0 μm and total exposure of 50 e^−^/Å^2^. We used a nominal magnification of ×81,000 or ×105,000, corresponding to images with a calibrated pixel size of 1.09 Å or 0.855 Å, respectively. The scale bar was added using ImageJ v1.53t after the raw images were binned by a factor of 4.

### Vaccine preparation

An appropriate amount of CPQ^21^ or CPQ^18^ was incubated with antigens at a mass ratio of CoPoP to total antigen of 3:1. The mixture was further incubated for 3 h at room temperature to allow the His-tagged antigen to bind to CoPoP. The final volume was adjusted to 50 µL with filtered PBS per mouse per injection. Other CoPoP liposomes were prepared in the same way. Vaccines were stored or transferred at 4 °C.

### Slot blot

Each sample was prepared to contain 10 ng of total protein in 200 µL for each slot. The microfiltration apparatus was assembled, and samples were filtered through a 0.2 µm nitrocellulose membrane. The membrane was blocked by PBS containing 5% BSA for 1 h at 37 °C, then washed twice with PBS. The following primary antibodies were diluted and incubated with each strip of membrane containing all samples: anti-H1 A/Brisbane/59/2007 (International Reagent Resource, Cat# FR-494), anti-H3 A/Victoria/361/2011 (International Reagent Resource, Cat# FR-1122), anti-B HA B/Brisbane/60/2008 (International Reagent Resource, Cat# FR-1337), anti-N1 A/Brisbane/59/2007 (International Reagent Resource, Cat# FR-941), anti-N2 A/Perth/16/2009 (International Reagent Resource, Cat# FR-1156), and anti-B NA hmAb (1092E10) which kindly provided by Dr. James Kobie. After another 1 h of incubation at 37 °C, all strips were washed 3 times with PBS, then incubated with HRP-linked anti-mouse or anti-human secondary antibody for 30 min at 37 °C. The membrane was washed three times and then imaged with the Bio-Rad ChemiDoc Imager.

### Ni-NTA binding test and SDS-PAGE

Ni-NTA magnetic beads (ThermoFisher, Cat# 88831) were used at 2 µL per 1 µg of protein. Beads were washed three times with PBS on a magnetic rack to remove storage buffer, then incubated with protein samples at room temperature. During incubation, beads were gently mixed by pipetting every 10 min for a total of three cycles. Following incubation, supernatants were collected, and the beads were resuspended in an equal volume of PBS. Both the supernatant and bead fractions were mixed with diluted 5× reducing sample buffer (ThermoFisher, Cat# 39000) and boiled at 95 °C for 5 min. After cooling to room temperature, samples were loaded onto 4–12% Bis-Tris protein gels (ThermoFisher, Cat# NP0321BOX) and electrophoresed for 30 min at a constant 200 V. Gels were rinsed twice with DI water, briefly heated, rinsed again, and then stained with Coomassie blue dye (Invitrogen, Cat# LC6065) for 10 min. Excess stain was removed by immersing gels in DI water for 1 h, and images were captured using a Bio-Rad GelDoc imager.

### Hemolysis assay

Whole blood from turkeys was obtained from Lampire Biological Labs. 5 mL of Turkey whole blood was transferred into a 15 mL centrifuge tube, and 10 mL of PBS was added. The mixture was centrifuged for 5 min at 300 × *g*, and the supernatant was discarded. The washing step was repeated twice. The blood sample was diluted with PBS and mixed with serially diluted free QS-18, free QS-21, CPQ^18^, or CPQ^21^ in a 96-well U-bottom plate for 30 min on the shaker at room temperature. The supernatant was transferred to a clear 96-well flat-bottom plate after centrifuging the 96-well U-bottom plate, and then the absorbance was measured at 540 nm to calculate the degree of hemolysis.

### In vivo toxicology

For mouse toxicology, the left footpads of 4–6 weeks old BALB/c mice (*n* = *4*) received one injection per 50 µL dose containing 1.8 µg total antigen, 5.4 µg CoPoP, 2.16 µg PHAD, and 2.16 µg QS adjuvant. The mice were kept under anesthesia with an oxygen supply throughout the injection process. One tablet of gabapentin was given as the treatment for post-anesthesia for one day. The footpad size was measured every 6 h for 24 h. For body weight monitoring, another group of 4-6-week-old BALB/c mice (*n* = *5*) received one intramuscular injection containing 1.8 µg total antigen, 5.4 µg CoPoP, 2.16 µg PHAD, and 2.16 µg QS adjuvant per 50 µL dose, and the body weight was measured daily. Blood was collected on day 10 for serum and CBC panel analysis.

The non-GLP exploratory toxicity study with New Zealand rabbits (a standard species for vaccine and adjuvant toxicology studies according to current FDA, ICH, and WHO guidelines on clinical evaluation of vaccines) was carried out at Inotiv, Inc. Three male and three female New Zealand white rabbits were assigned to each group. All animals received the following formulation once through intramuscular injection into the left lateral thigh under appropriate restraint: 30 µg QS18, 75 µg CoPoP, 30 µg PHAD3D6A per 0.5 mL dose; 30 µg QS21, 75 µg CoPoP, 30 µg PHAD3D6A per 0.5 mL dose; 50 µg QS18, 125 µg CoPoP, 50 µg PHAD3D6A per 0.5 mL dose; 50 µg QS21, 125 µg CoPoP, 50 µg PHAD3D6A per 0.5 mL dose; 125 µg CoPoP; 50 µg PHAD3D6A per 0.5 mL dose as vehicle; or sterile saline (0.9% Sodium Chloride). The injection site was evaluated using the Draize grading scale (0-4) for edema and erythema, where 0 indicated no reaction, and 4 indicated severe reaction. Clinical pathology, including coagulation, hematology, and chemistry, was performed at SD -6, SD 3, and SD 8. Hematology parameters were measured and calculated by the Siemens Advia 2120I hematology analyzer. Coagulation was measured by the instrumentation laboratory ACL Elite Pro coagulation analyzer. Serum concentrations of the clinical chemistry were measured by the Siemens Dimension Xpand chemistry analyzer. The mean and standard deviation were generated by Provantis^TM^ version 11 or greater at Inotiv, Inc. Rabbits were euthanized by intravenous sodium pentobarbital overdose (Euthasol^®^).

### Enzyme-linked immunosorbent assay (ELISA)

ELISA plates were coated with 100 ng/well of the corresponding antigen in coating buffer (5.3 g/L Na₂CO₃ and 4.2 g/L NaHCO₃ in DI water, pH 9.6) and incubated overnight. Serum was diluted 1:200 in PBS containing 0.1% Tween-20 (PBST) and then subjected to 10-fold serial dilutions for up to six dilutions. Plates were washed twice with PBST, blocked with PBST containing 2% BSA for 1 h at 37 °C, and then incubated with serially diluted sera for 1 h at 37 °C. After three washes with PBST, wells were incubated with HRP-conjugated anti-mouse secondary antibody (1:1000 dilution) for 30 min at 37 °C. Plates were washed five times, and TMB substrate was added for 10 min at room temperature. The reaction was stopped with 1 M HCl, and absorbance at 450 nm was measured using a plate reader.

### Hemagglutination inhibition (HAI) assay

For the hemagglutination inhibition (HAI) assay, 20 µL of serum was mixed with 100 µL PBS and 80 µL receptor-destroying enzyme (RDE II) and incubated overnight at 37 °C. RDE activity was inactivated by heating to 56 °C for 1 h. Treated sera were diluted to an initial 1:40 and further subjected to 2-fold serial dilutions up to 1:2560. Turkey red blood cells (RBCs) were prepared by centrifuging 5 mL of whole blood at 300 × *g* for 5 min, washing with PBS, and resuspending at a 10% dilution in PBS before storage at 4 °C. The working virus titer was determined by incubating serial dilutions of virus with RBCs. For HAI, 25 µL of diluted virus was mixed with 25 µL of serially diluted sera and incubated at 37 °C with gentle shaking for 30 min. Subsequently, 50 µL of diluted RBC suspension was added, and the mixture was incubated at room temperature for 30 min before assessing hemagglutination.

### Neuraminidase inhibition (NAI) assay

For the neuraminidase inhibition (NAI) assay, sera were processed as described in the HAI assay section. Plates were coated overnight at 4 °C with 100 µL of fetuin (25 µg/mL) in coating buffer and washed twice with PBST. Processed sera were subjected to two-fold serial dilutions in a separate plate and incubated with diluted viral stock. The mixtures were transferred to fetuin-coated plates and incubated for 18 h at 37 °C. Plates were washed five times and incubated with peanut agglutinin conjugated to HRP (PNA-HRP, 1 µg/mL in PBST) for 2 h at room temperature in the dark. After five additional washes, TMB substrate was added for 10 min, the reaction was stopped with 1 M HCl, and absorbance was measured at 450 nm.

### Mice immunization and influenza challenge

4–6 weeks old ICR or BALB/c mice were immunized intramuscularly with 50 µL of vaccine formulation containing QS-18- or QS-21-adjuvanted liposomes, followed by a booster dose on day 21, under appropriate restraint (1.8 µg total antigen, 5.4 µg CoPoP, 2.16 µg PHAD, and 2.16 µg QS adjuvant per 50 µL dose). Viral challenge and viral burden analysis were performed as previously described^23^. Briefly, on day 42 post-prime, mice were challenged intranasally with 50 µL of mouse-adapted H1N1, H3N2, or B-Vic influenza virus, continuously added in small drops, alternating nares, while anesthetized with 5% isoflurane in an O_2_ chamber. Animals were monitored daily for 14 days for body weight and clinical score (criteria including piloerection, labored breathing, hunched posture, lethargy, abnormal gait, and emaciation with greater than 10% weight loss), and those exhibiting a body weight loss greater than 25% were euthanized by cervical dislocation under deep anesthesia. Lung homogenates were collected on 4 or 7 dpi for lung viral load analysis. Briefly, mice were deeply anesthetized with 3% isoflurane in the 100% O_2_ chamber. Approximately 0.5 mL of blood was collected via the abdominal aorta for serum isolation. The lungs were harvested and homogenized using a Bullet Blender, and the homogenate was then clarified by centrifugation at 500 *g* for 5 min at 4 °C. The supernatant was then flash-frozen and stored at *−*80 °C for viral titers determination by plaque assay on Madin-Darby Canine Kidney (MDCK) cells. Serum was isolated by centrifuging at 2000 RPM for 20 min and stored at *−*80 °C.

### Ferret immunization and influenza challenge

A/Wisconsin/67/2022 (H1N1) in vivo challenge described in Fig. 7 with 13–15 weeks old Fitch ferrets (*n* = *6*) was performed by Bioqual, Inc. Briefly, ferrets received 6 µg of recombinant HA and NA of A/Wisconsin/67/2022 (H1N1), A/Darwin/6/2021 (H3N2), and B/Austria/1359417/2021 (B-Vic) formulated with 108 µg of CoPoP, 43.2 µg QS-21 or QS-18, and 43.2 µg PHAD-3D6A per 0.5 mL dose, on day 0 and day 21, through intramuscular injection under appropriate restraint. Ferrets were anesthetized by intramuscular injection (IM) of ketamine and dexmedetomidine before in-life procedures, including blood collection, viral challenge, and nasal lavage. Physiological parameters (pulse, respiration, pink mucous membrane) and anesthetic depth were monitored throughout the procedure. Following ketamine and dexmedetomidine anesthesia, animals received an IM dose of Antisedan^®^ to aid in recovery and returned to housing units for continued monitoring until completely recovered. Ferrets were deeply anesthetized by intramuscular injection with ketamine HCl (15 mg/kg) and dexmedetomidine (0.03 mg/kg) prior to terminal procedures. After nasal lavage and blood collection procedures, euthanasia was performed with Euthasol^®^ overdose via anterior vena cava or intracardiac injection, and euthanasia was confirmed by thoracotomy. Sera were shipped to the University at Buffalo for additional serological analysis.

### Ethics statement

All animal experiments were conducted in accordance with relevant institutional guidelines for the care and use of laboratory animals. Experiments described in Fig. 2, Fig. 4, Supplementary Figs. 2 and 3 were approved by the University at Buffalo Institutional Animal Care and Use Committee (IACUC). Experiments described in Fig. 3, Supplementary Fig. 4–8 were conducted at Inotiv, Inc. and approved by IACUC and found to be in accordance with provisions of the USDA Animal Welfare Act, the PHS Policy on Humane Care and Use of Laboratory Animals, and the US Interagency Research Animal Committee Principles for the Utilization and Care of Research Animals. Experiments described in Figs. 5 and 6 were approved by the Veterans Administration Western New York Healthcare System’s IACUC. Experiments described in Fig. 7 were conducted at Bioqual, Inc. and approved by IACUC.

## Supplementary information

npj Vaccines QS-18 SI

## Data Availability

All data supporting the findings of this study are presented in the main text figures and supplementary information. Data and statistical analysis were performed by GraphPad Prism (Version 10.1.1).
