# Supplementary material for: Quillaja saponaria fraction QS-18 as an adjuvant for liposomal seasonal influenza vaccines
Source: NPJ Vaccines. 2026 Apr 28;11:135. doi: 10.1038/s41541-026-01457-1 (PMC13328653; doi:10.1038/s41541-026-01457-1)
Supplement: Supplementary file 1 — npj Vaccines QS-18 SI [file 41541_2026_1457_MOESM1_ESM.pdf]

Supplemental Information: Quillaja Saponaria Fraction QS-18 as an Adjuvant for Liposomal Seasonal Influenza Vaccines

Qinzhe Li, Zachary Sia, Yuan Luo, Wei-Chiao Huang, Hilliard L. Kutscher, Haojun Zhu, Joaquin Ortega, Bruce A. Davidson, Jonathan F. Lovell

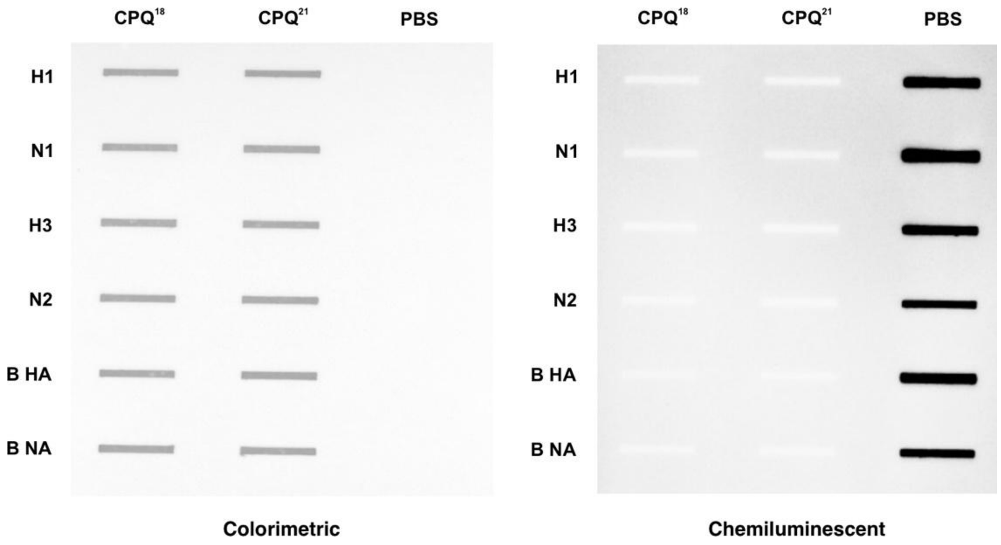

Supplementary Fig. S1. His-tag of antigens was not accessible to anti-His tag monoclonal antibody after coupling with CPQ[18] or CPQ[21] by slot blot assay.

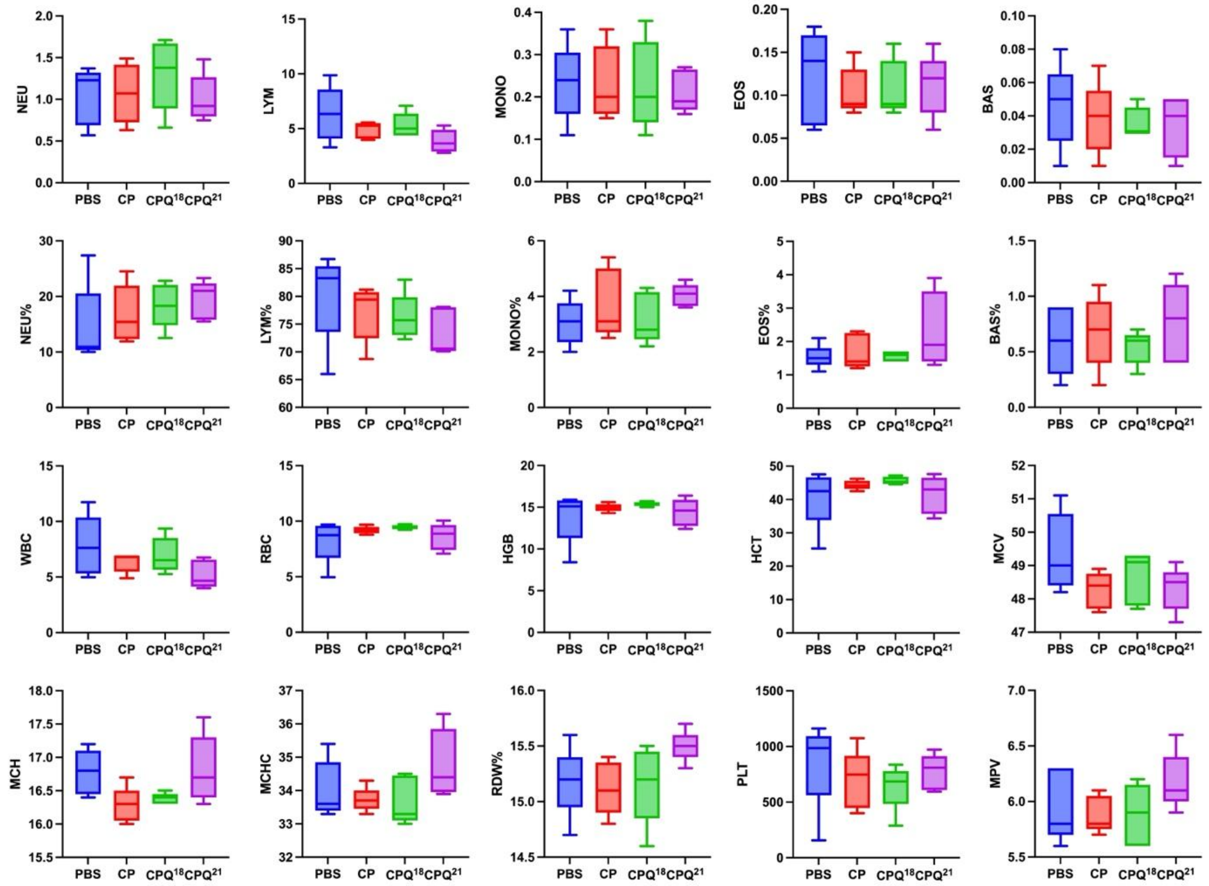

Supplementary Fig. S2. CBC panel after mice were immunized with CoPoP/PHAD QS21 or QS18 liposome.

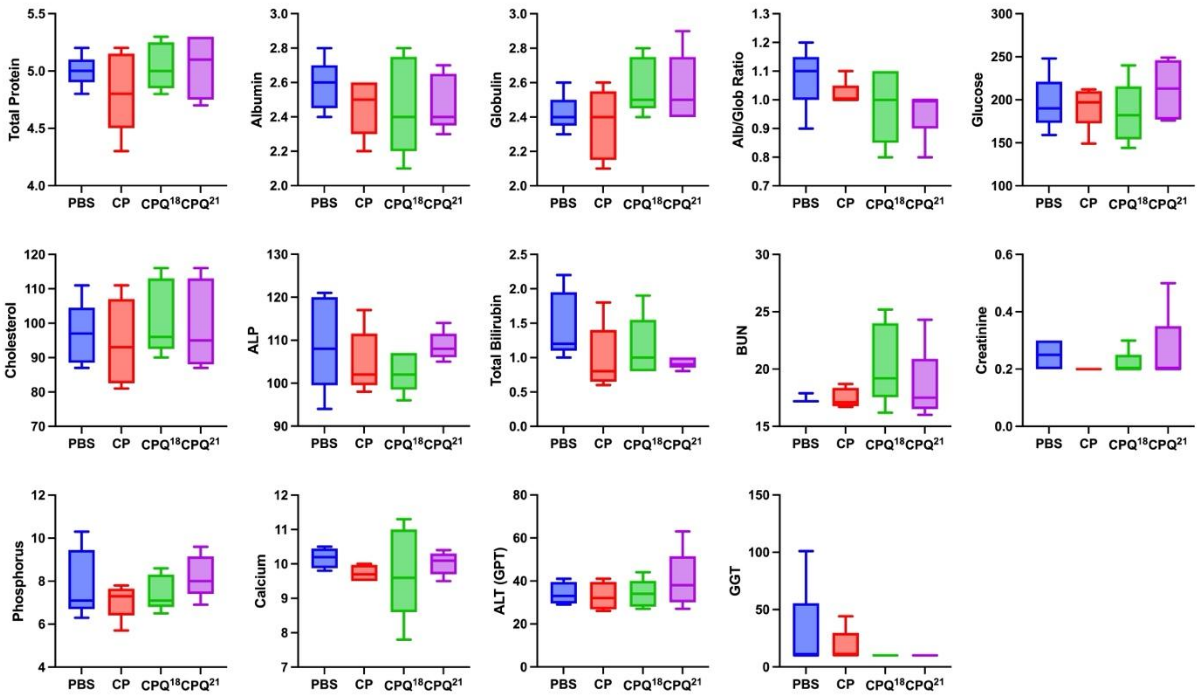

Supplementary Fig. S3. Serum panel after mice were immunized with CoPoP/PHAD QS21 or QS18 liposome.

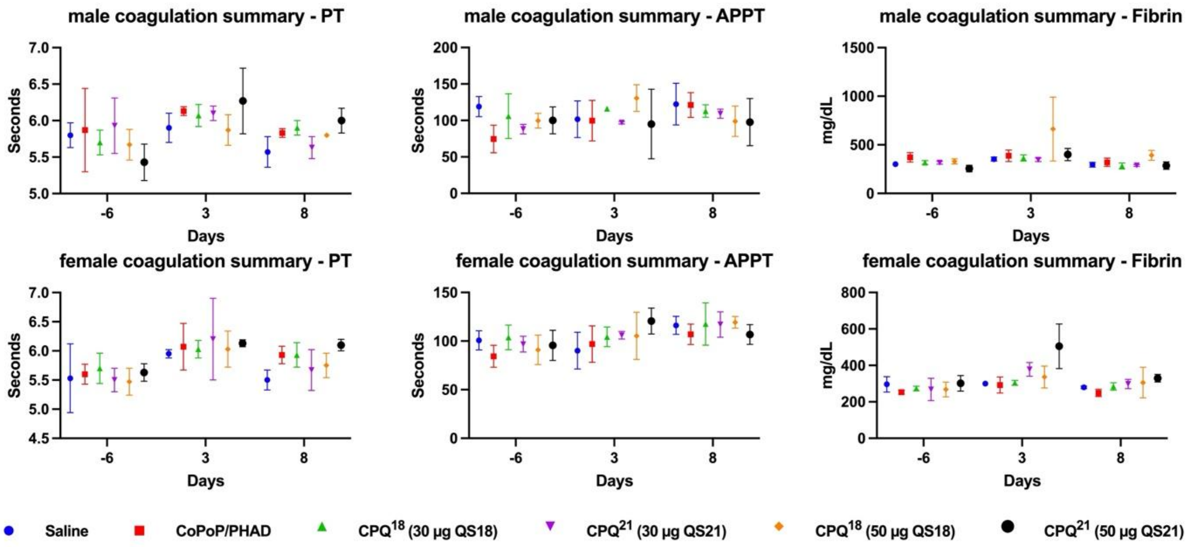

Supplementary Fig. S4. Coagulation of New Zealand rabbits.

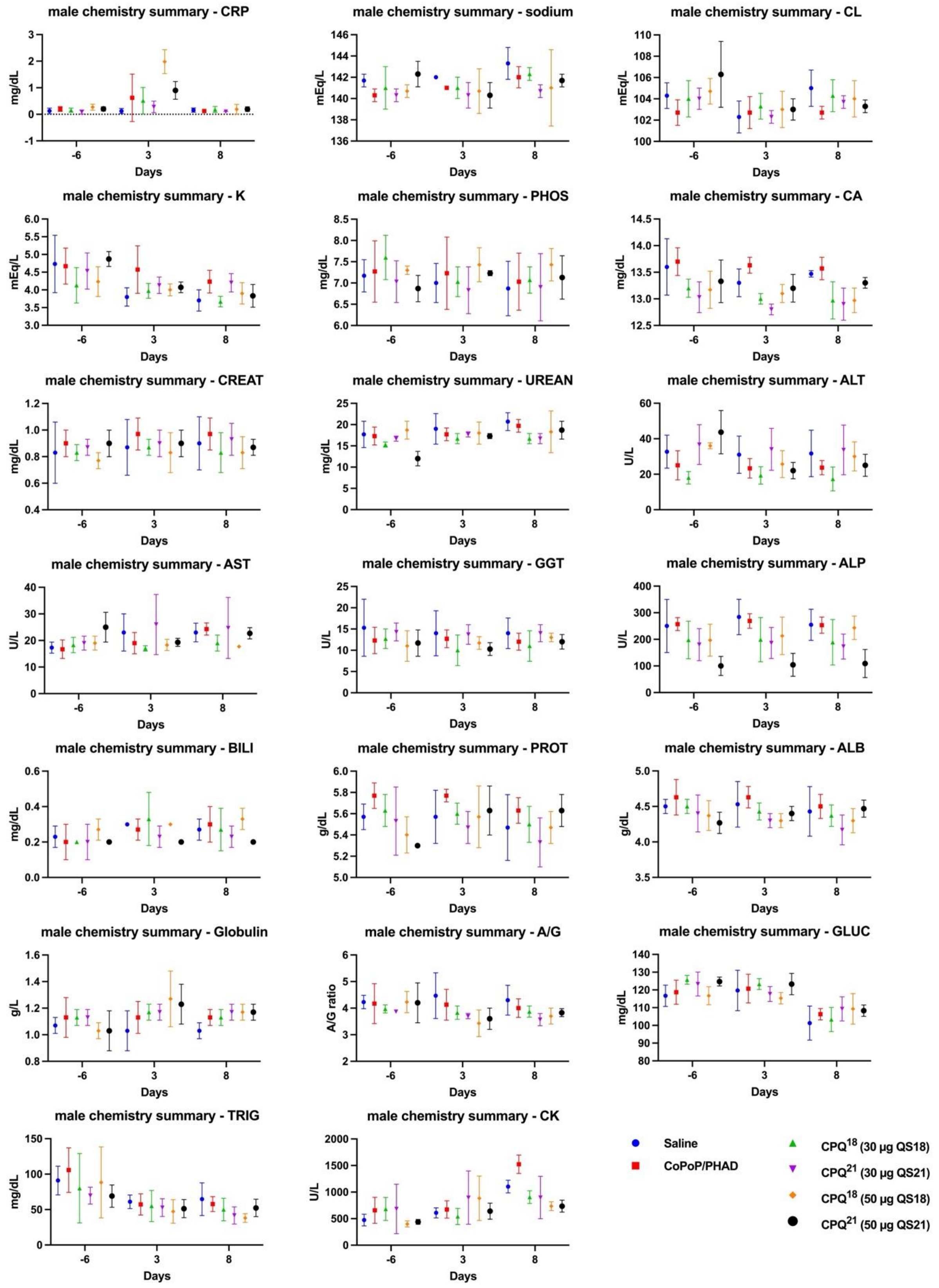

Supplementary Fig. S5. Chemistry panel of male New Zealand rabbits.

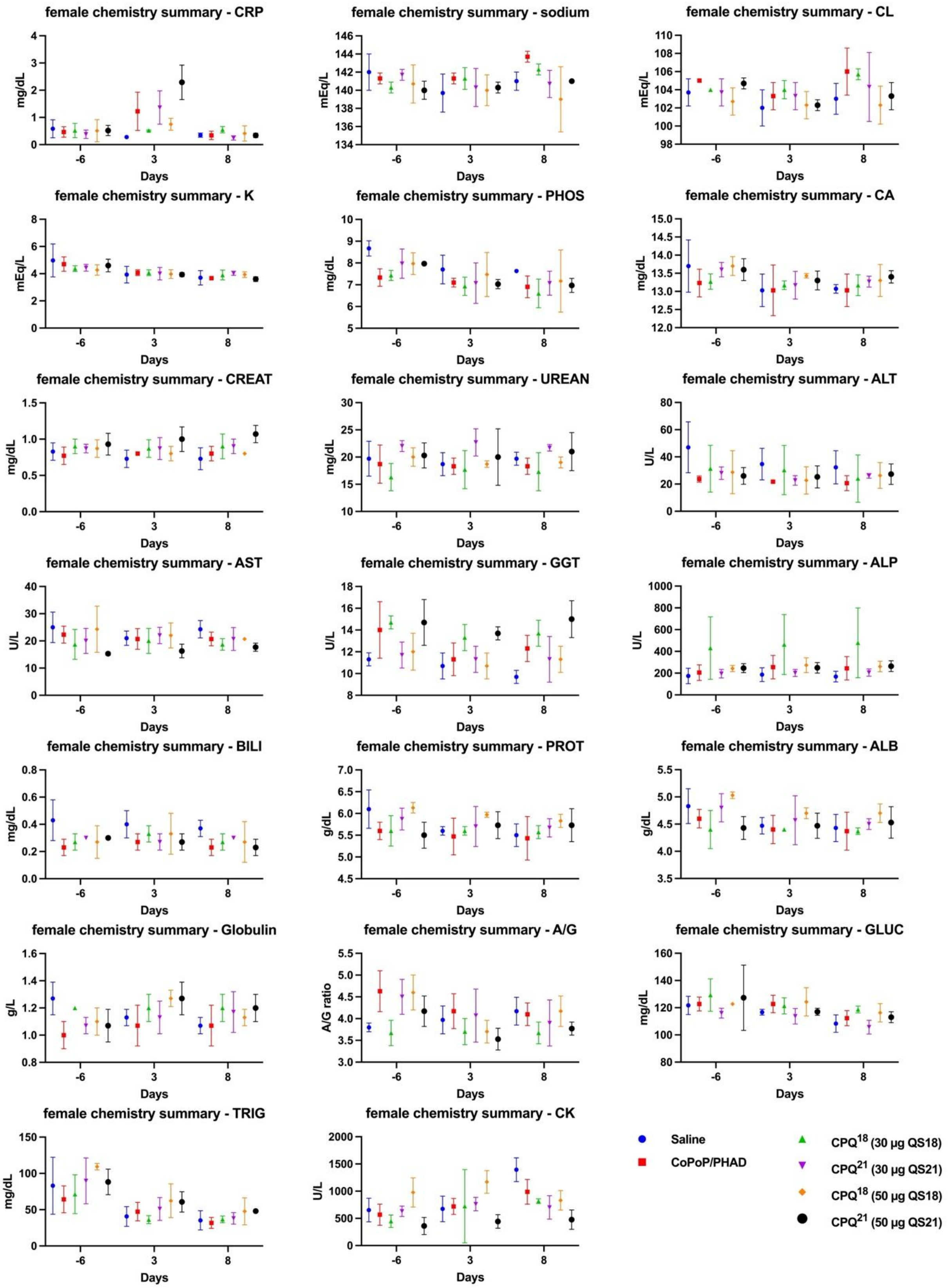

Supplementary Fig. S6. Chemistry panel of female New Zealand rabbits.

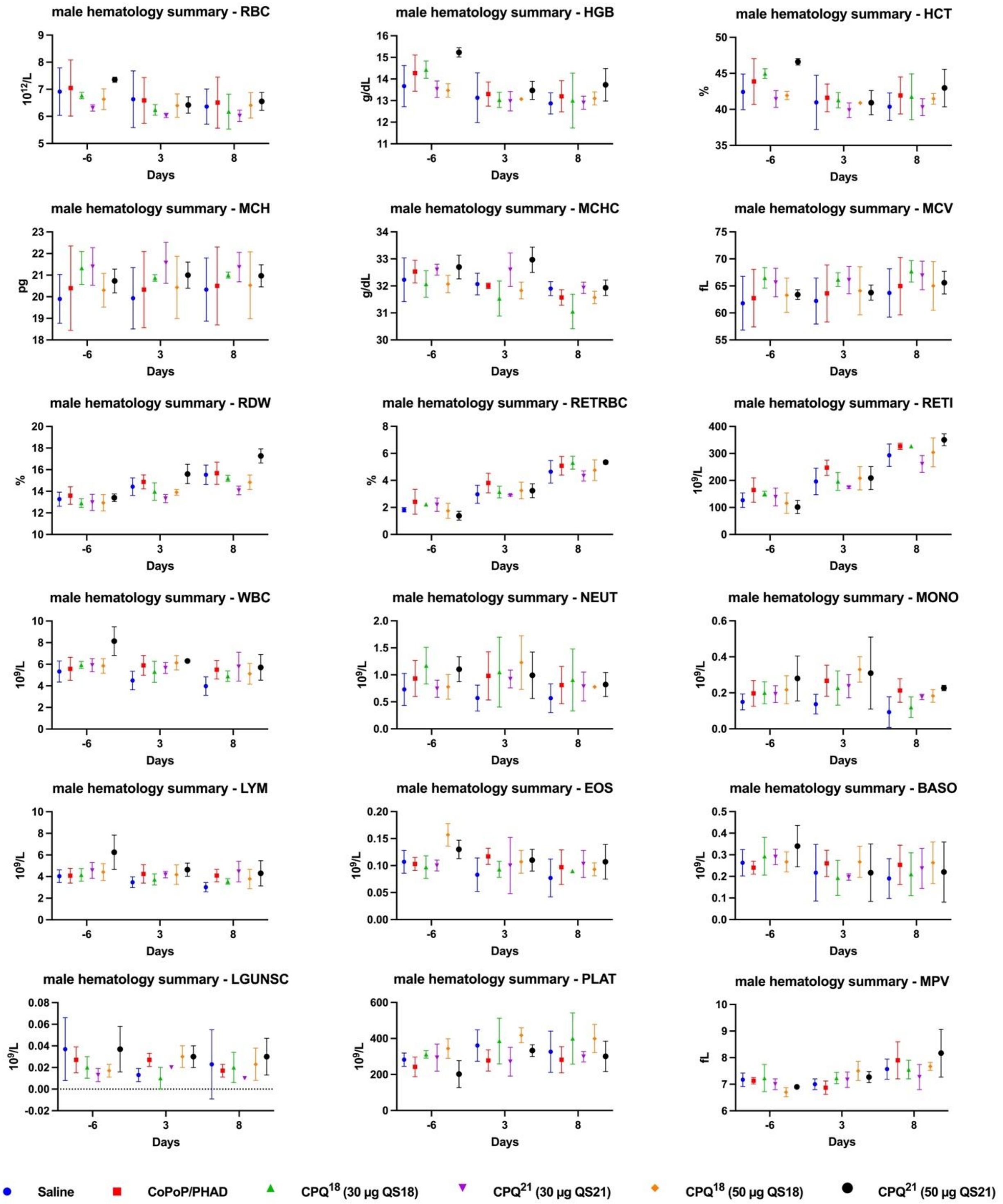

Supplementary Fig. S7. Hematology panel of male New Zealand rabbits.

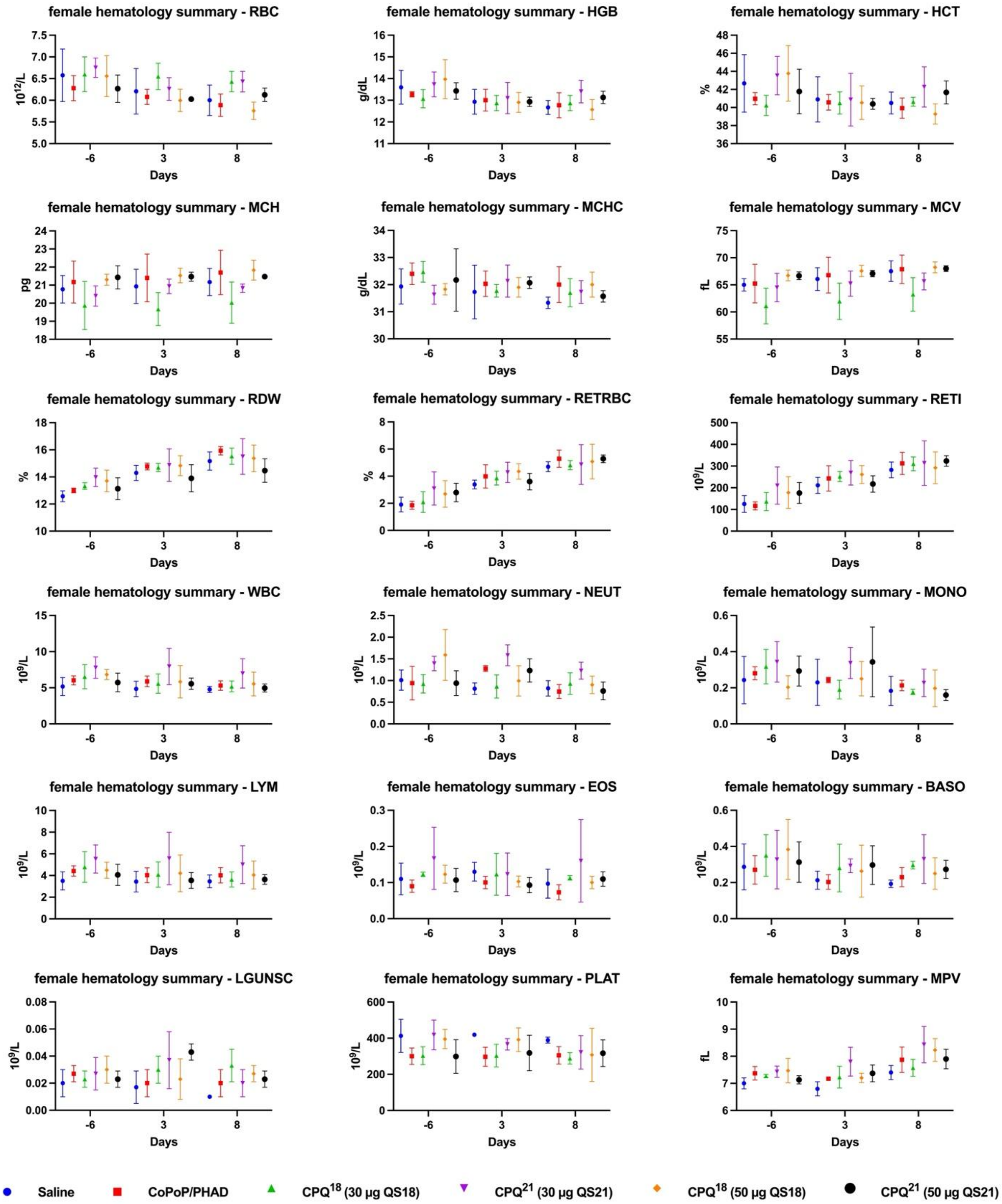

Supplementary Fig. S8. Hematology panel of female New Zealand rabbits.
